# Occupational Allergies

**DOI:** 10.1155/2011/519329

**Published:** 2011-07-21

**Authors:** Gordon Sussman, Donald Beezhold

**Affiliations:** ^1^Division of Allergy and Clinical Immunology, University of Toronto, Toronto, ON, Canada M5S 1A1; ^2^Allergy and Clinical Immunology Branch, The National Institute for Occupational Safety and Health, 1095 Willowdale Road, Morgantown, WV 26505, USA

## 

Occupational immune diseases are new emerging illnesses that affect workers in industrialized societies. Occupational exposures to substances in the workplace environment can cause inflammation, allergy, or other potentially detrimental immune responses. Personal exposure to a variety of chemicals can exacerbate immune diseases such as contact dermatitis as well as respiratory diseases including rhinitis, asthma, and hypersensitivity pneumonitis. 

Next to illnesses due to repeated traumatic injury, contact dermatitis is the second most commonly reported occupational illness. It can prevent individuals from performing job-related tasks or preclude working altogether. Occupationally related contact dermatitis is a significant public health burden with combined direct annual cost estimates of up to $1 billion in the USA for medical costs, workers compensation, and lost time from work. 

Respiratory morbidity is also a significant burden to public health leading to lost productivity. Prevalence rates for occupational rhinitis are significant, varying by occupation between 5% and 65% and costing an estimated $593/year/employee due to productivity losses. Conservative estimates made by the American Thoracic Society in 2003 estimate that 15% of chronic obstructive pulmonary disease and asthma cases were work related and cost approximately $7 billion in lost productivity in the USA With the changing work environment, new occupational hazards continue to emerge which require immunologic characterization. In order to reduce the morbidity and mortality associated with these illnesses, it is critical that we identify the allergens and understand the immunological mechanism by which they exacerbate immune-mediated respiratory and dermal diseases. Specific understanding of mechanism has direct implications in developing appropriate intervention and prevention strategies.

Occupational allergy can be stratified into high-molecular-weight-allergen and low-molecular-weight-allergen mediated responses. Different immunologic mechanisms mediate allergic reactivity to these occupational allergens as highlighted in this issue by Talini et al. High-molecular-weight (HMW) allergens (typically proteins) induce type I hypersensitivity responses or typical allergies by inducing IgE antibodies which lead to a continuum of symptoms including rhinitis (rhinosinusitis, conjunctivitis), hives, asthma, and life-threatening anaphylaxis. Patients with HMW-allergen-induced asthma show a greater frequency and severity of the early-phase response but are less likely to demonstrate a late-phase response. Occupational outbreaks of reactions to HMW allergens can occur episodically and can be severe and life altering for those affected. These allergies can affect large numbers of easily identified workers in specific industries which can reach epidemic proportions such as latex allergy and Baker's asthma. It can present in a less-well-defined population or as local occurrences such as agricultural or food processors exposed to soy, sea foods, pollens, molds, and so forth. Research areas include identification and characterization of high-molecular-weight occupational allergens. Using fungal enzymes as a prototypic HMW occupational allergen, Green et al. describe some of the characterized fungal enzyme allergens and discuss monitoring and avoidance strategies. Characterization of HMW allergens includes using proteomics, molecular techniques and generating recombinant allergens, and producing monoclonal antibodies for the development of immunoassays and improved detection of the allergens in the workplace.

Low-molecular-weight allergens (typically chemicals) induce type 4 hypersensitivity reactions by inducing allergen-specific T lymphocytes which can mediate contact dermatitis reactions as well as sensitizations that can lead to severe asthma such as isocyanates (auto painters) and trimellitic acid. Patients with LMW-allergen-induced asthma are more likely to demonstrate a late-phase airway response. The review by Anderson et al. describes the identification of low-molecular-weight allergens in the laboratory using the local lymph node assay to determine whether new chemicals being introduced can cause workplace sensitizations as well as testing various components to identify the specific sensitizer and potentially nonsensitizing replacements. They examined the effects of chemical exposure on immune function using selected assays from a comprehensive tiered approach. This can be used in detecting toxic effects following chemical exposure (in rodents) as adopted by the National Toxicology Program. The utility of analyzing potential replacement chemicals is highlighted by the study of Johnson et al. where the chemical *ortho*-phthalaldehyde (OPA) has been recommended as a substitute for glutaraldehyde as a sterilant in the healthcare industry. Their laboratory evidence suggested that the replacement of the chemical OPA is also a strong sensitizer. Characterization of the biochemical and immune mechanisms by which chemicals become allergens (haptenization) is described in a comprehensive review by Chipinda et al. Developing new methods for screening chemicals for potential sensitizers helps to build better models by which we predict whether chemicals are allergens. Yucesoy et al. describe new studies aimed at identifying occupationally sensitized individuals and understanding the genetic profile associated with sensitizing/anaphylactic agents. 

It is important to improve our basic science knowledge and understanding of occupational allergies and their pathogenesis. If we are able to identify potential allergens, before clinical symptoms are observed, employers can take necessary precautions to minimize or eliminate their employee's exposure.

